# Catchment-scale export of antibiotic resistance genes and bacteria from an agricultural watershed in central Iowa

**DOI:** 10.1371/journal.pone.0227136

**Published:** 2020-01-10

**Authors:** Timothy P. Neher, Lanying Ma, Thomas B. Moorman, Adina C. Howe, Michelle L. Soupir

**Affiliations:** 1 Department of Agricultural and Biosystems Engineering, Iowa State University, Ames, Iowa, United States of America; 2 National Laboratory for Agriculture and the Environment, USDA-ARS, Ames, Iowa, United States of America; Purdue University, UNITED STATES

## Abstract

Antibiotics are administered to livestock in animal feeding operations (AFOs) for the control, prevention, and treatment of disease. Manure from antibiotic treated livestock contains unmetabolized antibiotics that provide selective pressure on bacteria, facilitating the expression of anti-microbial resistance (AMR). Manure application on row crops is an agronomic practice used by growers to meet crop nutrient needs; however, it can be a source of AMR to the soil and water environment. This study in central Iowa aims to directly compare AMR indicators in outlet runoff from two adjacent (221 to 229 ha) manured and non-manured catchments (manure comparison), and among three catchments (600 to 804 ha) with manure influence, no known manure application (control), and urban influences (mixed land use comparison). Monitored AMR indicators included antibiotic resistance genes (ARGs) *ermB*, *ermF* (macrolide), *tetA*, *tetM*, *tetO*, *tetW* (tetracycline), *sul1*, *sul2* (sulfonamide), *aadA2* (aminoglycoside), *vgaA*, and *vgaB* (pleuromutilin), and tylosin and tetracycline resistant enterococci bacteria. Results of the manure comparison showed significantly higher (p<0.05) tetracycline and tylosin resistant bacteria from the catchment with manure application in 2017, but no differences in 2018, possibly due to changes in antibiotic use resulting from the Veterinary Feed Directive. Moreover, the ARG analysis indicated a larger diversity of ARGs at the manure amended catchment. The mixed land use comparison showed the manure amended catchment had significantly higher (p<0.05) tetracycline resistant bacteria in 2017 and significantly higher tylosin resistant bacteria in 2017 and 2018 than the urban influenced catchment. The urban influenced catchment had significantly higher *ermB* concentrations in both sampling years, however the manure applied catchment runoff consisted of higher relative abundance of total ARGs. Additionally, both catchments showed higher AMR indicators compared to the control catchment. This study identifies four ARGs that might be specific to AMR as a result of agricultural sources (*tetM*, *tetW*, *sul1*, *sul2*) and optimal for use in watershed scale monitoring studies for tracking resistance in the environment.

## Introduction

The animal agricultural industry is the largest consumer of antibiotics worldwide, used for the control, prevention, and treatment of bacterial diseases, as well as growth promotion in livestock animals [1]. In the period between 2010 and 2030, global antibiotic usage rates in agriculture are predicted to increase by at least 67% and nearly double in developing countries where legislative enforcement on antibiotic use is lacking [2]. The United States in 2017 added new regulations to the Veterinary Feed Directive, changing medically important antibiotics from over the counter to requiring a veterinary prescription [3], in an effort to minimize Anti-Microbial Resistance (AMR) in agriculture. The directive should reduce medically important antibiotic usage, however, antibiotics are known to persist in the soil environment [4]. Manure from antibiotic treated livestock is known to contain unmetabolized antibiotics that provide selective pressure on bacteria and facilitates the spread of AMR to the environment [5, 6]. AMR is a natural mechanism in bacteria [7, 8] used to thwart antibiotic bactericidal properties, rendering treatments ineffective. It is enriched when antibiotic and bacteria interactions are increased, such as in the gut of treated animals, in manure storage facilities, and in soils amended with manure [9]. AMR is a global public health concern, as AMR can pass to pathogenic bacteria and potentially cause an incurable infection. It is estimated that 700,000 deaths worldwide are currently attributed to AMR per year, and if action is not taken to halt the spread, the effects of AMR could cost the global economy a massive 100 trillion USD between now and 2050 [10].

The main source of bacteria and AMR in agriculture is from manure amendments to soil. Manure application as fertilizer is commonly practiced in global agriculture [11] to improve soil fertility and to properly dispose of animal waste. Manure is rich in essential nutrients necessary for plant growth and also provides physical improvements to soils such as increases in water holding capacity, regulation of soil temperature, and improved aggregate stability [12]. Fields are typically amended with manure in early spring before planting or in late fall, ideally after soil temperatures are below 10 degrees Celsius to limit nutrient losses and prevent ammonia volatilization. However, manure contains high levels of potentially pathogenic bacteria, and manure application on row crops is a primary source of fecal contamination to receiving downstream waters in agricultural landscapes [13]. Furthermore, animal production trends have shifted towards larger scale animal feeding operations (AFOs) to maximize production efficiency. Areas of the Upper Midwest of the U.S.A. have high densities of AFOs integrated in the landscape, and these can be breeding grounds for AMR development due to increased interaction between antibiotics and bacteria [14].

Bacteria transport in the environment is closely associated with water flow [15, 16]. Agricultural water export in the American Midwest is a combination of surface flow formed from overland runoff, and subsurface drainage. In the Great Lakes and Corn Belt states, 37% of cropland is artificially drained by tile drainage [17] to improve water infiltration, allowing machinery access to fields and improved root zone aeration for optimal crop growth among other benefits [18]. Although this has led to some of the most productive cropland in the world, tile-drainage has changed the natural hydrology of the region by increasing water flow to downstream catchments, consequently expediting the transport of pollutants to receiving waterbodies [19, 20]. Tile-drainage may also facilitate the export of bacteria and AMR as found in previous studies [21, 22, 23]. Because water transports bacteria potentially containing AMR, the importance of monitoring runoff from manure amended lands is crucial to understanding the factors that most closely influence the potency and export of bacteria and AMR.

While the export of fecal indicator bacteria (FIB) from agricultural landscapes has been well studied [24, 25, 26], there are few studies that have been conducted on antibiotic resistance genes (ARGs) export at plot and watershed scales [23, 27, 28]. Missing from the literature is monitoring of AMR from paired catchments with and without manure application. Therefore, the goal of this study was to characterize the extent of AMR at the catchment scale in an agricultural watershed in central Iowa. The objectives are to monitor tile and surface water for total fecal indicator bacteria, antibiotic specific resistant fecal indicator bacteria, and ARGs. Comparisons are made between two catchments with differences in manure application practices, and among three larger catchments with differences in manure application and urban influences. This study provides further evidence on the impact of manure application practices on the detection of AMR in agricultural runoff and helps identify more robust indictors for AMR monitoring.

## Materials and methods

### Site description

The Black Hawk Lake (BHL) watershed is located in western Iowa on the border of Sac and Carroll Counties and along the western edge of the Des Moines Lobe, the furthest extent of glacial formation from around 12,000 years ago [29]. The area is characterized by poorly drained soils and consisting of glacial till derivatives. The watershed is 5,324 hectares and is dominated by loams and clay loams formed from glacial till. Land use in the watershed includes 74.6% row crops, 6.7% grass/hay, 5.8% wetlands, 1.9% timber, and 11% other [30]. Two municipalities lie in the watershed: Lake View, population 1,142, lies in the northwest region and Breda, population 483, is situated at the southernmost end of the watershed at the headwaters (2010 census). Much of the cropland in the BHL watershed is tile-drained due to the naturally hydric and poorly drained soils native to the Des Moines Lobe. It is assumed that all surface flow channels in the watershed are influenced by tile-drainage discharge.

**Fig 1:**
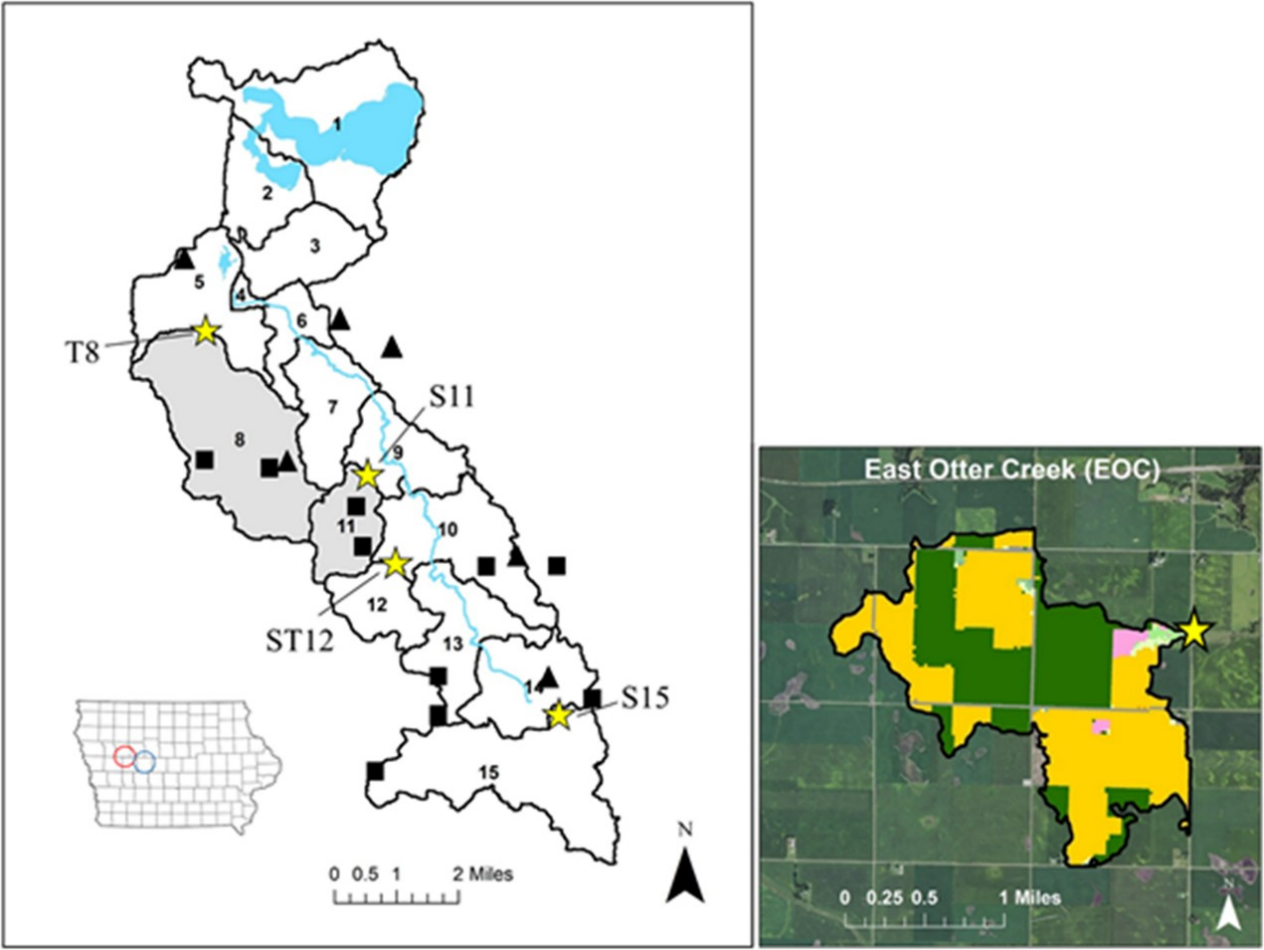
Monitored locations in the Black Hawk Lake watershed and the control catchment. Black Hawk Lake watershed (left; red circle) monitoring locations marked by yellow stars. The grey shaded regions indicate monitored catchments known to receive manure. Triangles indicate beef/dairy feedlots and squares indicate swine confinements. The East Otter Creek (EOC) catchment (right; blue circle) and monitoring location. Satellite imagery source: USDA NAIP Imagery.

Swine and cattle animal feeding operations (AFOs) are distributed throughout the watershed (Fig 1) documented during a windshield survey in 2017. There are ten swine confinements and six cattle lots within and just outside of the watershed border, all of which contain less than 2,000 animal units (IOWA GEODATA). Manure generated by AFOs in the watershed is mainly applied in the west-central region (S1 Fig). The historical application of manure in the watershed is unknown, although it is assumed manure has been applied broadly. Furthermore, Best Management Practices (BMPs) have been implemented within the watershed at land-owner discretion, intended to reduce nutrient and sediment losses to downstream waters. Consequently, the distribution of BMPs are uneven throughout the watershed. The most common BMPs in the BHL watershed are terraces, grass waterways, cover crops, no-till, strip-till, perennial native grass plantings (CRP), and nutrient management plans.

The primary watershed was delineated into fifteen catchments with independent drainage areas for study (Fig 1). Catchments 8 and 11 are known to receive manure (Personal communication, T.J. Lynn Dec. 14th, 2017) and have low mixed BMP coverage (< 30%) by at least one type of BMP and consisting of at least two AFOs within the drainage area. Catchment 12 has high mixed BMP coverage (87.5%) by at least one type of BMP and no manure application. This catchment has two monitoring locations at the outlet; a surface outlet and a tile discharge outlet. Catchment 15 has low mixed BMP coverage (21.4%) by at least one type of BMP and does not receive manure application, but drains effluent from the town of Breda, population 409, along with two AFOs that may contribute to bacteria inputs. Further hydrologic characterization can be found in [19].

### Study design

Monitoring of fecal indicator bacteria and AMR in the BHL watershed occurred from May 2017 through early December 2018. The locations monitored within the BHL watershed included four catchment outlets, S15 (42°11'53.8"N 94°58'31.1"W), ST12 (42°13'29.6"N 95°00'45.8"W), S11 (42°14'21.9"N 95°01'03.1"W), and T8 (42.261103, -95.055583) (Fig 1). The “S” and “T” denote surface flow and tile-drain discharge, respectively. The number refers to the catchment from which the discharge originated. Catchment 12 has both a tile (T) and surface (S) outlet at the monitoring site, therefore is named ST12. A catchment outside of the watershed and in the Des Moines Lobe was added to the study in 2018 to serve as a no-manure control, East Otter Creek (EOC), (42°00'00.9"N 94°27'16.7"W) to be compared to data collected at S15 and T8. (See S1 Table for detailed catchment properties). Permissions to access sites S11 and ST12 were obtained from the Sac County Secondary Roads Department. Site T8 access was granted by the local landowner on the land which the tile-main discharges. All other sites were accessed off of public roadsides.

Samples were analyzed using phenotypic techniques to quantify FIB resistant to tylosin and tetracycline and genotypic techniques to quantify select antibiotic resistance genes. Tetracycline and tylosin were chosen for the phenotypic analysis because they are classified as medically important antibiotics, both used to treat swine and cattle as well as having a link to human medicine. Tetracyclines are used to treat various types of bacterial infections in humans [31], and tylosin is in the macrolide antibiotic class that shares cross-resistance with other macrolides used in clinical settings such as erythromycin used to treat skin and respiratory tract infections [32]. The genotypic analysis quantifies the antibiotic resistance genes *ermB*, *ermF* (macrolide), *tetA*, *tetM*, *tetO*, *tetW* (tetracycline), *sul1*, *sul2* (sulfonamide), *aadA2* (aminoglycoside), *vgaA*, and *vgaB* (pleuromutilin). These ARGs were chosen to represent a wide variety of antibiotic classes because the antibiotic usage in the surrounding AFOs of this study were generally unknown. Macrolides, tetracyclines, sulfonamides, and aminoglycosides are broadly used in livestock production [3], and pleuromutilins are unregulated under the VFD and may become a popular choice among livestock owners.

### Sample collection

Samples were collected at each site in 1 L high-density polyethylene (HDPE) bottles every two weeks in sampling year 2017 and every week in sampling year 2018. The following were the total number of samples collected at each site (2017 and 2018, respectively): S11 (16, 33); ST12 (15, 33); S15 (19, 33); T8 (20, 33); EOC (0, 23). Surface water samples were collected at the mid-point of each stream channel in the highest velocity flow. Tile-drainage samples were taken directly from the tile discharge. Each sampling location was sampled on the same day to minimize influence of outside factors and to reduce variability between sites such as changes in temperature, precipitation, or UV exposure. All grab samples were preserved on ice and then transferred to the Water Quality Research Lab at Iowa State University and stored at 4°C for no longer than 24 hours before filtering and storage.

Flow was measured at each location and on each sampling date. Manual measurements were taken using the float method as described in [33]. Discharge measurements were taken at tile-drained sites by measuring the length of time to fill a known volume. Beginning in March 2018, a Model 2100 Current Velocity Meter (Swoffer Instruments, Inc.) was used to measure velocity for the manual flow measurements at each site location. In addition, select catchment sites were equipped with ISCO model 750 Area Velocity Module (sites T8, S11, and T12) or an ISCO Model 720 Submerged Probe Module (site S12) throughout the entirety of the study and this flow data is used preferentially to the manual flow measurements at respective sites.

### Phenotypic plate count analysis

Grab samples from the field were analyzed for total enterococci, total *E*. *coli*, tylosin resistant and tetracycline resistant enterococci by the membrane filtration technique as described by [34]. Each sample was filtered individually through a 0.45 μm sterile disc filter wetted with phosphate-buffered saline (PBS) buffer. Selective Agar used for the analysis was mEnterococcus agar (Difco, Detroit Michigan), mEnterococcus agar with 16 mg/L tetracycline (Sigma-Aldrich, St. Louis, MO), mEnterococcus agar with 35 mg/L tylosin (Sigma-Aldrich, St. Louis, MO), and mTEC agar (Difco, Detroit Michigan). Each antibiotic infused agar was made so that the concentration of active antibiotic in each plate was slightly above the resistance breakpoint concentration to select for antibiotic resistant bacteria growth [35]. The mEnterococcus plates were incubated at 35°C for 48 hours and the mTEC plates were incubated at 35°C for two hours and 44.5°C for 22 hours in a hot water bath. Plates were counted for CFUs (Colony Forming Units). Final counts were reported as CFU/100mL. Samples were diluted and re-plated if 250 or more CFU were counted, indicating TNTC (Too numerous to count). Bacterial enumerations determined to be TNTC halfway through incubation were re-analyzed no more than 48 hours after collection. If plates were deemed TNTC, but not re-plated in time, then they were treated as 250 CFU/plate and reported as CFU/100mL based on the dilution factor. These results are included in the statistical analysis of the Wilcoxon Rank-Sum Test described subsequently. Because of the ranking system of the test, the measurement represents a sample consisting of high bacterial concentration that should not be disregarded.

### DNA extraction

A portion of each water sample (250 mL) was filtered through a 0.25 μm sterile filter within 24 hours of sampling and stored at -80°C until DNA extraction. Subsequent DNA extraction from the filters were performed using the standard protocol of MagAttract PowerWater DNA/RNA Kit (Qiagen, USA) and Eppendorf epMotion 5075 automated robot (Eppendorf, USA). DNA concentrations were measured following the standard protocol of ThermoFisher Scientific Quant-iT ^TM^ dsDNA Assay Kit, high sensitivity. The DNA was then stored at -80°C until further analysis.

### Genotypic qPCR analysis

All environmental water samples were analyzed for the 16S rRNA bacterial gene abundance and a range of genes coding for antibiotic resistance for common antibiotics used in swine and cattle production. These genes include *ermB*, *ermF* (macrolide), *tetA*, *tetM*, *tetO*, *tetW* (tetracycline), *sul1*, *sul2* (sulfonamide), *aadA2* (aminoglycoside), *vgaA*, and *vgaB* (pleuromutilin) (Table 1). The qPCR analysis was performed on four runs on the Takara (previously Wafergen) SmartChip Realtime PCR System (Wafergen Inc. USA) in the 144 sample x 36 target format at Michigan State University Research Technology Support Facility (East Lansing, MI). Each run included three control samples filled with PCR water.

**Table 1:** List of primers used in the study [36].

Gene	Forward Primer (5'-3')	Reverse Primer (5'-3')	Mechanism
**16S rRNA**	CCTACGGGAGGCAGCAG	ATTACCGCGGCTGCTGGC	N/A
***ermB***	GAACACTAGGGTTGTTCTTGCA	CTGGAACATCTGTGGTATGGC	protection
***ermF***	TCTGATGCCCGAAATGTTCAAG	TGAAGGACAATTGAACCTCCCA	protection
***tetM***	GGAGCGATTACAGAATTAGGAAGC	TCCATATGTCCTGGCGTGTC	protection
***tetA***	CTCACCAGCCTGACCTCGAT	CACGTTGTTATAGAAGCCGCATAG	efflux
***tetO***	CAACATTAACGGAAAGTTTATTGTATACCA	TTGACGCTCCAAATTCATTGTATC	protection
***tetW***	ATGAACATTCCCACCGTTATCTTT	ATATCGGCGGAGAGCTTATCC	protection
***sul1***	GCCGATGAGATCAGACGTATTG	CGCATAGCGCTGGGTTTC	protection
***sul2***	TCATCTGCCAAACTCGTCGTTA	GTCAAAGAACGCCGCAATGT	protection
***aadA2***	ACGGCTCCGCAGTGGAT	GGCCACAGTAACCAACAAATCA	deactivate
***vgaA***	GGAAGCTATAGAGGCGTTTGAATC	CCGAAGGTTCAATACTCAATCGAC	efflux
***vgaB***	CAGCCGGATTCTGGTCCTT	TACGATCTCCATTCAATTGGGTAAA	efflux

Gene abbreviation, forward and reverse primers used in this study, and associated antibiotic resistance mechanism.

#### Standard curve method

Four out of the twelve gene targets (16S rRNA, *ermB*, *ermF*, and *tetM*) were run with an associated standard curve for gene quantification and reported as copies 100 mL^-1^. Therefore, these four genes have unique LOQs per qPCR run based on the results of each respective standard curve (Table 2). Each LOQ per run for the four genes is based on the most dilute standard included in the linear portion of the standard curve. The limit of detection (LOD) is reported as a detected value by the qPCR analysis that does not meet the LOQ threshold.

**Table 2:** LOQ and LOD for the four selected genes in the standard curve method.

Gene	Run	Date Range	LOQ (copies/100mL)	Total samples	% samples > LOQ	LOQ > % samples > LOD	% sample < LOD
**16S rRNA**	2017	4/20–12/19	330,142	125	98.4	0.0	1.6
	2018–1	3/27–7/11	49,577	117	94.0	4.3	1.7
	2018–2	7/18–10/2	1,598,059	117	37.6	41.9	20.5
	2018–3	10/11–11/27	248,521	91	71.4	16.5	12.1
***ermB***	2017	4/20–12/19	1,774	125	7.2	24.8	68.0
	2018–1	3/27–7/11	1,466	117	9.4	13.7	76.9
	2018–2	7/18–10/2	18,482	117	0.0	7.7	92.3
	2018–3	10/11–11/27	298	91	14.3	0.0	85.7
***ermF***	2017	4/20–12/19	2,744	125	0.0	24.0	76.0
	2018–1	3/27–7/11	2,727	117	4.3	17.1	78.6
	2018–2	7/18–10/2	2,211	117	0.9	6.8	92.3
	2018–3	10/11–11/27	4,949	91	0.0	14.3	85.7
***tetM***	2017	4/20–12/19	3,258	125	14.4	36.0	49.6
	2018–1	3/27–7/11	2,858	117	11.1	28.2	60.7
	2018–2	7/18–10/2	2,734	117	6.9	14.5	78.6
	2018–3	10/11–11/27	20,065	91	2.2	28.6	69.2

The 2017 samples were measured in a single analytical run while the 2018 samples required 3 separate runs. The Date Range column indicates the timeframe of samples that were collected and ran in each corresponding run. Each LOQ is run specific and samples below LOQ were reported as zero for analysis.

#### Relative abundance method

Because of our limited access to gene standards to make standard curves for every analyzed gene, a relative abundance analysis was done to compare the Ct-value of all the ARGs to the Ct-value of the total bacteria gene, 16S rRNA, using the relationship, as previously described [37].

$${Relative\;abundance=2}^{-\Delta Ct},\;\mathrm{where}\;\Delta Ct=Ct_{\mathrm{sample}}-Ct_{16S\;\mathrm{rRNA}}$$

For this analysis method, the threshold cycle value (Ct) of 28 was used as the limit of quantification (LOQ) for the relative abundance analysis, and any sample with a Ct-value above the threshold was considered below the LOQ and was assigned a value of zero. The stringent LOQ of Ct < 28 was chosen for this study to ensure higher confidence in the results and to minimize false positives.

All primer sets and samples were run in triplicate. The gene copy and relative abundance analyses are separate and all genes for the relative abundance analysis including 16S rRNA, *ermB*, *ermF*, and *tetM* follow the same LOQ (Ct<28) requirement instead of calculated standard curve LOQ from the gene copy analysis for consistency between methods. Quantified gene copy ARGs were normalized to the bacteria conserved gene 16S rRNA.

### Polar organic chemical integrative sampler (POCIS)

Runoff water at select sampling locations were analyzed with POCIS to detect for antibiotics isolated by extraction methods for macrolides, tetracyclines, and sulfonamides. The sampling outlet locations supplemented by POCIS were from two catchments that received manure application (S11 and T8) and two that did not receive manure application (ST12 and EOC). The POCIS were deployed at these select sampling locations to supplement our watershed analysis assessment that these catchments were amended with manure.

The extraction and analysis procedure were modified from the protocols outlined by [4]. The POCIS manifold was disassembled, then the hydrophilic-lipophilic balance (HLB) coated membranes were rolled and inserted into 50 mL Teflon centrifuge tubes with 25 mL of methanol. The centrifuge tubes were shook overnight at room temperature and then the membranes were removed and the solutions were centrifuged at 5,000 x g to pellet the HLB. Then 10 mL subsamples were evaporated to 0.5 mL and then brought up to a volume of 2 mL with ammonium acetate. After waiting 20 minutes, the solution was filtered through a 0.2 μm nylon filter into an HPLC vial and analyzed by LC-MS. The evaporation step and LC-MS analysis are described by [4].

### Statistical analysis

All statistical analyses were completed using JMP Pro 14 version 14.1.0. The non-parametric Wilcoxon Rank-Sum Test was used to test for significant differences between catchments with differing manure management, and among catchments with urban influence and manure application. Resulting p-values less than 0.05 (p<0.05) between two groups were classified as significantly different. Sampling years 2017 and 2018 are analyzed separately. Site comparisons include a manured comparison between manured site S11 versus non-manure site ST12, and a mixed land use comparison among manured T8 versus urban influenced S15 a non-manured and non-urban influenced control site, East Otter Creek (EOC). Three types of data were collected and used in the Wilcoxon Rank-Sum Test for comparisons: Phenotypic plate counts (CFU/100mL), gene quantification (copies/100mL), and relative abundance (unitless). 1.) Phenotypic plate count measurements include total enterococci and total *E*. *coli* comparisons. Percent tetracycline and tylosin resistant enterococci were compared among catchments and were calculated as the CFU/100mL tetracycline/tylosin count divided by the total enterococci count in the sample water multiplied by 100. 2.) The gene quantification data (copies/100mL) were from those four genes with associated standard curves (16S rRNA, *ermB*, *ermF*, *tetM*). Those four genes were compared individually among the catchments to test for differences between catchments. 3.) The relative abundance data from the qPCR assay for each of the eleven ARGs were grouped into a single dataset called total relative abundance for each catchment for comparison.

All data below limit of quantification were assigned a value of zero and included in statistical analyses unless otherwise stated.

The surface and tile-drainage flow pathways at ST12 were analyzed separately and results were combined flow-weighted by individually multiplying each concentration by its respective flow, then summing these products and dividing by the combined total flow. The combined result for each AMR indicator at ST12 was then used in statistical comparisons with catchment S11.

## Results

### Manure comparison

**Fig 2:**
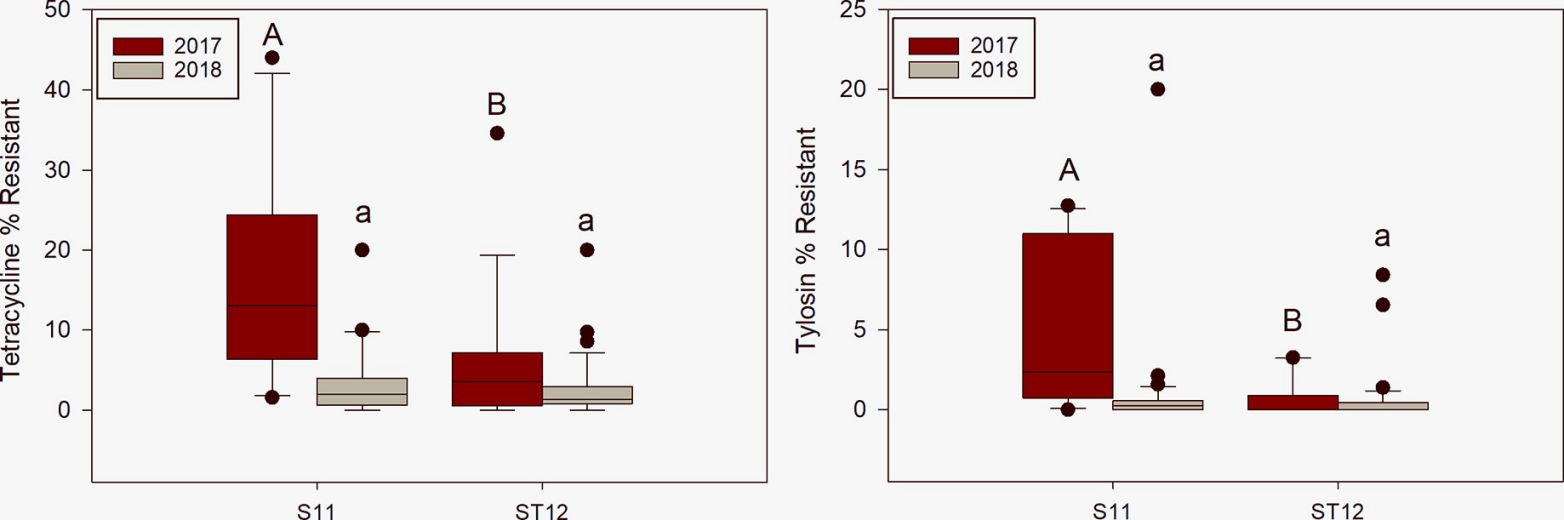
Box-plots of tetracycline and tylosin percent resistant enterococci for the manure comparison. The middle line on the box-plot represents the median while the upper quartile of the box represents the 75^th^-percentile and the lower quartile the 25^th^-percentile. The whiskers denote the range of the quartiles and outliers are marked as dots. The letters above each plot represent significant differences based on the Wilcoxon Rank-Sum Test. Capital letters are associated with 2017 and lower-case letters are associated with 2018. Differing letters mean significant difference between groups (p<0.05).

Total enterococci concentrations compared at manured catchment S11 versus non-manured catchment ST12 revealed that S11 had significantly higher (p = 0.0291) total enterococci concentrations in catchment outlet water during 2017 but no significant difference in 2018. Total *E*. *coli* concentrations between sites were not different in 2017, but S11 contained significantly higher (p = 0.0360) concentrations in 2018. Likewise, the catchment scale comparison of the resistant enterococci showed significantly higher tetracycline (p = 0.0060) and tylosin (p = 0.0033) resistance at S11 in 2017, but no significant differences in 2018 (Fig 2).

In 2017, we observed significantly higher (p = 0.0003) 16S rRNA in catchment outlet water of S11 than at ST12, and no significant difference between the two catchments in 2018. The ARGs between catchments showed no difference in ARGs with standard curves or total relative abundance between sites in 2017, however, in 2018 there was significantly higher *tetM* (p = 0.0113) copies 100 ml^-1^ in outlet waters from manured catchment S11 than from non-manured catchment ST12.

**Fig 3:**
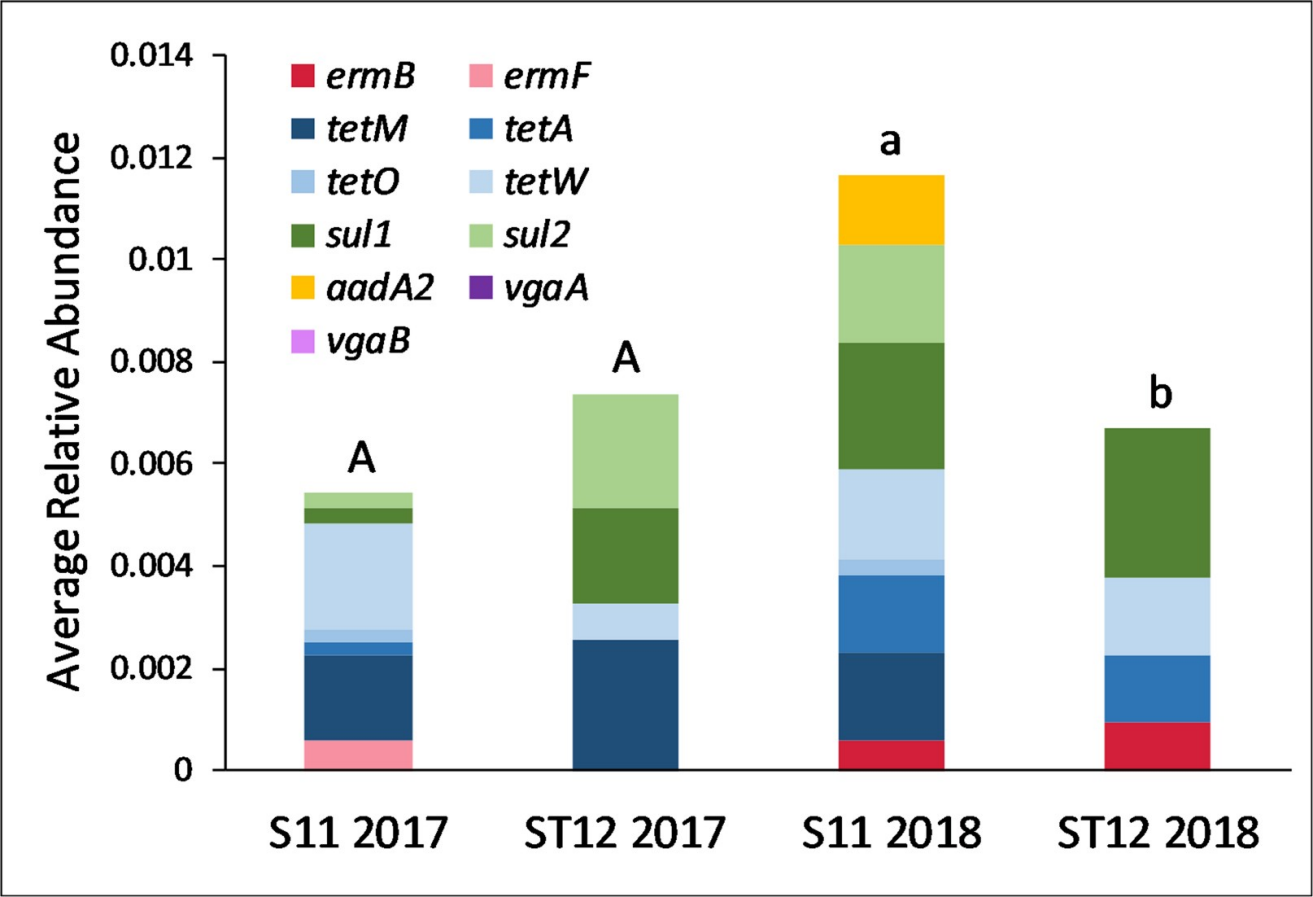
Average relative abundance from each ARG for the manure comparison. The letters above each plot represent total relative abundance significant differences based on the Wilcoxon Rank-Sum Test. Capital letters are associated with 2017 and lower-case letters are associated with 2018. Differing letters mean significant difference between groups.

The catchment outlet water at each site through both years consisted of ARGs determined through the relative abundance analysis. Of the eleven ARGs quantified, site S11 contained a larger diversity of ARGs than ST12 (Fig 3), more than likely due to manure application practices and the assumed varying antibiotics administered to livestock. The total relative abundance comparison between sites showed no difference in 2017, however, 2018 data showed S11 contained significantly greater (p = 0.0302) total relative abundance than ST12 in catchment effluent.

### Mixed land use comparison

The site comparison of urban influenced catchment S15 versus manured catchment T8 showed that S15 had significantly higher (p = 0.0136) enterococci concentrations in outlet waters in 2017 and in 2018. The control site outside of the BHL watershed, East Otter Creek (EOC), had significantly higher enterococci concentrations than both T8 (p<0.0001) and S15 (p = 0.0049) in 2018. Catchment discharge water at EOC was not collected in 2017. The urban influenced catchment S15 had significantly higher concentrations of *E*. *coli* than the manured catchment T8 in both 2017 (p = 0.0300) and 2018 (p<0.0015). The control catchment had significantly higher (p = 0.0077) concentrations of total *E*. *coli* than T8 and no significant difference between EOC and S15 in 2018.

**Fig 4:**
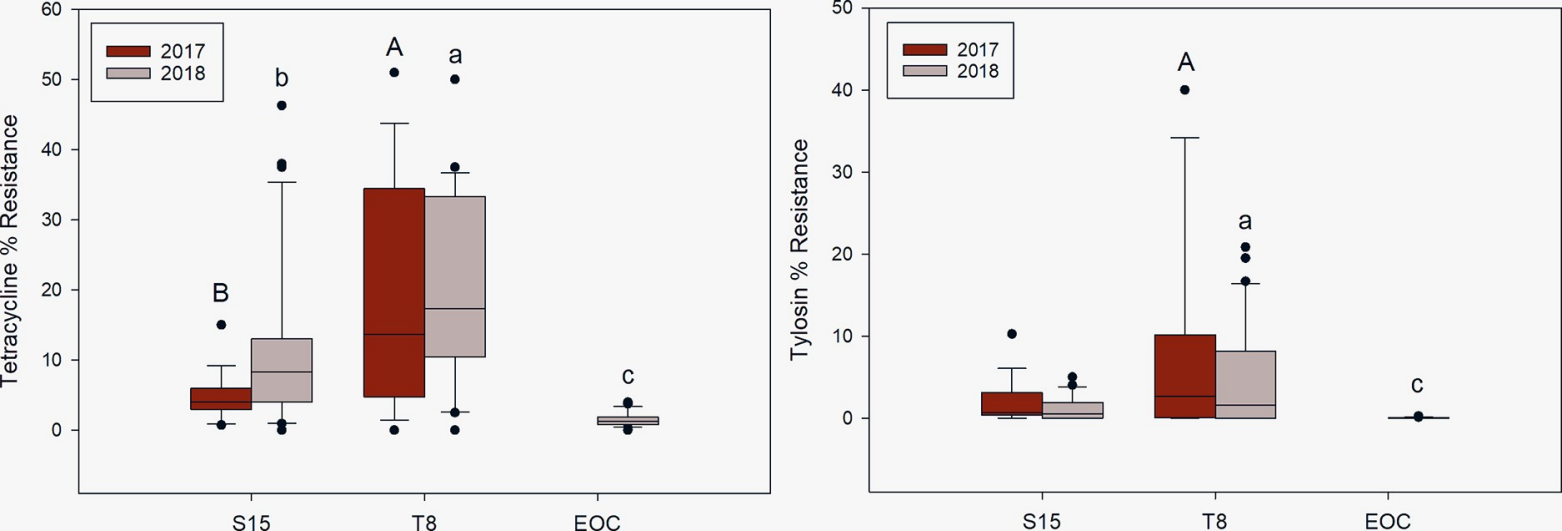
Box-plots of tetracycline and tylosin percent resistant enterococci for the mixed land use comparison. The middle line on the box-plot represents the median while the upper quartile of the box represents the 75^th^-percentile and the lower quartile the 25^th^-percentile. The whiskers denote the range of the quartiles and outliers are marked as dots. The letters above each plot represent significant differences based on the Wilcoxon Rank-Sum Test. Capital letters are associated with 2017 and lower-case letters are associated with 2018. Differing letters mean significant difference between groups.

The comparison of tetracycline resistant enterococci between the manured catchment T8 and the urban influenced catchment S15 showed significantly higher tetracycline resistance in T8 outlet waters in both 2017 (p = 0.0050) and 2018 (p = 0.0454) (Fig 4). The site comparison showed that catchments T8 and S15 both had significantly higher (p<0.0001) tetracycline resistance than the control catchment outside of the watershed. The comparison of tylosin resistant enterococci between the two BHL sites showed no significant difference in 2017, however, T8 had significantly higher (p = 0.0133) tylosin resistance in 2018. These two catchments both showed significantly higher (p = 0.0017) tylosin resistant enterococci when compared to the similarly sized control site EOC.

Catchment S15 effluent contained significantly higher 16S rRNA than catchment T8 in both 2017 (p<0.0001) and 2018 (p = 0.0130). Out of the three quantified ARGs *ermB*, *ermF*, and *tetM*, only *ermB* showed a significant difference in the median concentration of resistance gene copies between catchments S15 and T8. The *ermB* concentration at S15 was significantly higher in 2017 (p = 0.0236) and 2018 (p = 0.0162) than at T8 (Table 3). Catchment S15 contained the highest observed *ermB* in drainage waters. Additionally, catchment T8 contained spikes of *tetM* in catchment waters in 2018 with a median of samples above LOQ at 1,776 copies 100 mL^-1^ compared to median *tetM* copies of 340 copies 100 mL^-1^ at S15.

**Table 3:** Detection frequency and median copies/100mL above LOQ.

Year	Gene	Parameter	Site Location				
			Group 1		Group 2		
			S11	ST12	T8	S15	EOC
**2017**	**16S rRNA**	% > LOQ	100	94.4	100	95.0	-
		Median of % > LOQ (copies/100mL)	334,712*	149,425	83,620	443,048*	-
	***ermB***	% > LOQ	6.3	<LOQ	20.0	36.8	-
		Median of % > LOQ (copies/100mL)	194	<LOQ	250	257*	-
	***ermF***	% > LOQ	<LOQ	<LOQ	<LOQ	5.3	-
		Median of % > LOQ (copies/100mL)	<LOQ	<LOQ	<LOQ	61	-
	***tetM***	% > LOQ	12.5	6.7	20.0	26.3	-
		Median of % > LOQ (copies/100mL)	481*	173	737	212	-
			**S11**	**ST12**	**T8**	**S15**	**EOC**
**2018**	**16S rRNA**	% > LOQ	54.5	75.8	63.6	75.8	65.2
		Median of % > LOQ (copies/100mL)	47,210	112,607	52,302	243,617*	134,857
	***ermB***	% > LOQ	9.1	3.0	3.0	27.3	<LOQ
		Median of % > LOQ (copies/100mL)	552	111	286	412*	<LOQ
	***ermF***	% > LOQ	6.1	3.0	3.0	6.1	<LOQ
		Median of % > LOQ (copies/100mL)	532	0	0	565	<LOQ
	***tetM***	% > LOQ	18.2	<LOQ	27.3	24.2	<LOQ
		Median of % > LOQ (copies/100mL)	589	<LOQ	1776	340	<LOQ

The frequency of qPCR detection and median concentration of selected antibiotic resistance genes in stream water of the Blackhawk Lake Watershed above LOQ.

*Significantly greater between catchments in each group based on the Wilcoxon Rank-Sum Test. The statistical analysis includes zeros.

**Fig 5:**
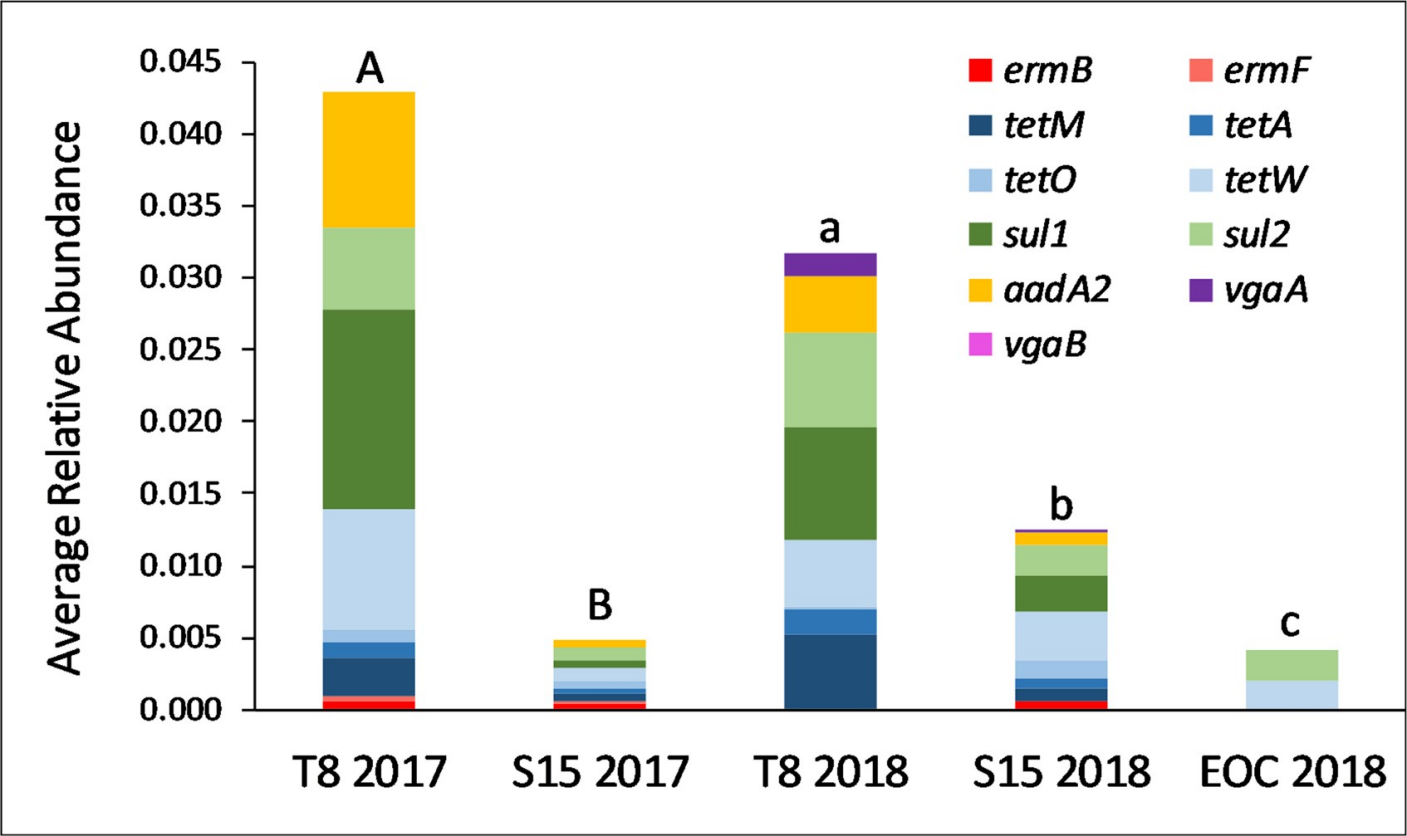
The average relative abundance from each ARG for the mixed land use comparison. The letters above each plot represent total relative abundance significant differences based on the Wilcoxon Rank-Sum Test. Capital letters are associated with 2017 and lower-case letters are associated with 2018. Differing letters mean significant difference between groups.

The manured catchment T8 consistently contained significantly greater total relative abundance in drainage effluent than the urban influenced S15 in both 2017 (p<0.0001) and 2018 (p = 0.0355). The control comparison showed catchments T8 and S15 had significantly higher (p<0.0001) total relative abundances than EOC (Fig 5).

### Antibiotic detection

The three measured antibiotics: macrolides, tetracyclines, and sulfonamides, were detected in all monitored runoff water from manured sites S11 and T8, and from non-manured sites ST12 and EOC. Tetracycline was detected in one out of ten and three out of ten samples from non-manured ST12 and EOC, respectively. Macrolides and sulfonamides were detected in all samples analyzed. The data summary can be found in the supporting material (S2 Table).

## Discussion

The manure comparison suggests that manure application influences bacteria and AMR indicators in catchment outlet waters. Total enterococci was significantly higher at the manured catchment in 2017 and total *E*. *coli* was significantly higher in 2018. This result agrees with previously observed findings of manure amendments increasing bacteria concentrations downstream [38, 39, 40], though contradicts the findings of [24] who observed no differences between concentrations of enterococci in drainage waters from plots treated with swine manure and plots free of manure. The catchment without manure application consistently showed lower measured AMR indicators in outlet waters than the catchment with manure application. However, differences between these two catchments may be levels of best management practices (BMPs) implemented by landowners, and the extent of tile-drainage, which may or may not influence the presence of AMR indicators in outlet waters. Little research has studied the effects of BMPs on bacteria export [41, 42], and even fewer on BMPs to reduce AMR in agriculture [43]. Tile-water on the other hand has been observed to contain higher concentrations of resistance constituents than in surface water and could also provide insight into the observation of higher total relative abundance at tile-drainage driven T8 than at mainly surface drained S15 [23]. Speculations for this may be the persistence of extracellular ARGs in the soil matrix [44, 45, 46], favorability of cell growth and decay in tile-lines, or the rapid transport of water containing AMR indicators to drainage discharge [44, 47]. Surface water samples from all catchment outlets are assumed to have inputs from upstream tile-discharge, but the extent is unknown.

Manure applied catchment T8 outlet water contained the highest mean relative abundance and most frequently detected ARGs of all sites in the two-year study. The most frequently detected ARG was *tetW*. The ARG *vgaA* was only detected at T8 and S15 but rarely and *vgaB* was not detected at all in 2018. The *vga* genes confer resistance to tiamulin, an antibiotic not regulated by the VFD and known to persist in animal manure [48] and when expressed, can confer cross resistance to macrolides [49]. Low detection of tiamulin resistance genes are expected, as previous studies on in vitro resistance development have shown low potential for success for resistance to fully develop [50, 51], as resistance develops in a slow step-wise fashion [52]. Alternatively, failure to detect tiamulin resistance could result from either poor amplification by the primers used in this study or the antibiotic is not used in sufficient quantity to generate detectable resistance genes.

The total relative abundance comparison between urban influenced S15 and manure applied T8 showed that T8 had significantly greater total relative abundance in both sampling years. Additionally, both catchments showed significantly greater total relative abundance than the non-manured control catchment outside of the main watershed. This follows similar observances from [28] who conducted a study in Southwestern British Columbia that compared ARGs from different watersheds each dominated separately by agriculture, human, or the natural environment and concluded that *sul1* and *sul2* were more prevalent in agriculture dominated watersheds while *tetW* was equally prevalent in agricultural and human dominated areas. These findings were reflected in this study as the manured catchment T8 had higher detections and average relative abundance of *sul1* and *sul2* than the urban influenced catchment S15. S15 and T8 shared almost equal detection frequency and average relative abundance of *tetW*.

The challenge in AMR monitoring in environmental systems is knowing whether the detected AMR indicators are simply part of the natural ecosystem [53] or a product of human impact, whether from agriculture or urbanization. This study progresses this knowledge gap by identifying ARGs associated with manure applied catchments which are concurrently undetectable in non-manure applied catchments. Out of all the ARGs analyzed in this study, those that are optimal for watershed scale monitoring in agricultural systems are *tetM*, *tetW*, *sul1*, and *sul2*. These genes are present in higher abundance at the outlet of manure applied catchments. This is supported by congruent studies such as aforementioned [28] who related *sul1* and *sul2* to agricultural systems, and a study by [54] who associated *tetW* with AFOs in a large scale river basin in Colorado. Moreover, The USEPA conducted a contiguous study on AMR genes from stream and river water and reported a positive relationship between *sul1*, urbanization, and agriculture [55].

The ARG copy 100 mL^-1^ mixed land use comparison showed differences in *ermB* concentration at S15 were significantly higher (p = 0.0063) than at T8. It is possible that this detection of *ermB* could originate from urban influence [56] such as leaky wastewater infrastructure, as *ermB* is a class of macrolide that shares cross resistance with other MLSB drugs used in human medicine [57]. However, the waste water treatment facility for the municipality is downstream of the monitoring location. It is also possible that some crop fields in catchment S15 might be amended with manure that originated from macrolide treated livestock, as two swine confinements straddle the catchment border. Additionally, catchment T8 contained spikes of *tetM* in catchment waters in 2018. These spikes occurred during low to normal flow rates in the growing season, suggesting other unknown drivers may have influenced the increases in export of ARGs, for example the lag-time between ARG source deposition and subsequent mobility within the soil/water matrix [9]. Another possibility might be a sudden release of active antibiotics previously held inert by soil may create a localized rapid production of ARGs in bacteria that are then transported to catchment outlets through normal flow conditions [58]. Regardless, both catchments contributed to AMR in comparison to the control site outside of the watershed. The control catchment’s high concentrations of FIBs may be due to alternative sources such as geese or grazing cows in pastureland.

The broad antibiotic detection by POCIS at all selected catchments is not surprising given the ubiquity of antibiotic use in agriculture and human health as well as the persistence of antibiotics in soil [4]. The goal of the antibiotic analysis was to provide supplementary evidence of manure application practices in each catchment based on the presence versus absence of antibiotics. The assumptions of manure amendments in catchments for this study are based on present conditions and supplemented by communications with the Black Hawk Lake watershed coordinator, GIS manure maps, and AFO locations. Historical manure application practices in each catchment is unknown and could influence the detected antibiotic levels at all catchments as well as influence AMR indicators.

## Conclusions

The scale of this study allowed for better confidence in comparisons of monitored AMR indicators due to differences in manure application practices or land use factors. At the catchment scale, we observed differences between catchments within the watershed that differed in manure application practices and urban influence. The manure comparison results may imply that manure application significantly increases resistance indicators in catchment outlet waters. The mixed land use comparison results may imply that urban influences may contribute more to FIB in outlet waters, and manured fields may contribute more ARG to the environment. However, both catchments contributed to AMR in comparison to the control site. Moreover, the manure associated ARGs *tetM*, *tetW*, *sul1*, and *sul2*, should be considered for watershed monitoring studies and are a good indication of human induced spread of AMR through agriculture, especially when all four are detected together.

Considering the present study findings, future studies should focus on ways to address the spread of bacteria and AMR such as manure mitigation prior to field application, in addition to in-field mitigation strategies to improve ARG attenuation in soils to limit the degree of ARG pollution to waterways. Although the scale of this study is relatively small, confounding factors between catchments are possible such as the amount and types of BMPs implemented, the extent of tile-drainage contributions, and urban influences. Further research should investigate the potential for specific BMPs to reduce ARG export from manure applied fields. Additionally, future studies could expand to include human specific fecal indicator gene targets to better assess urban contributions of AMR to the environment.

## Supporting information

S1 Fig Black Hawk Lake watershed manure extent map. Provided by T.J. Lynn, BHL Watershed Coordinator.
(TIF)

S1 Table Landscape and soil properties of monitored catchments. *Cropland Data Layer 2018, **Web Soil Survey.
(PDF)

S2 Table Antibiotic concentrations (pg/pocis) from select monitoring locations. (PDF)

S1 Data (XLSX)

S2 Data (XLSX)

S3 Data (XLSX)
