# Supplementary figures and images for: Catchment-scale export of antibiotic resistance genes and bacteria from an agricultural watershed in central Iowa
Source: PLoS One. 2020 Jan 10;15(1):e0227136. doi: 10.1371/journal.pone.0227136 (PMC6953785; doi:10.1371/journal.pone.0227136)

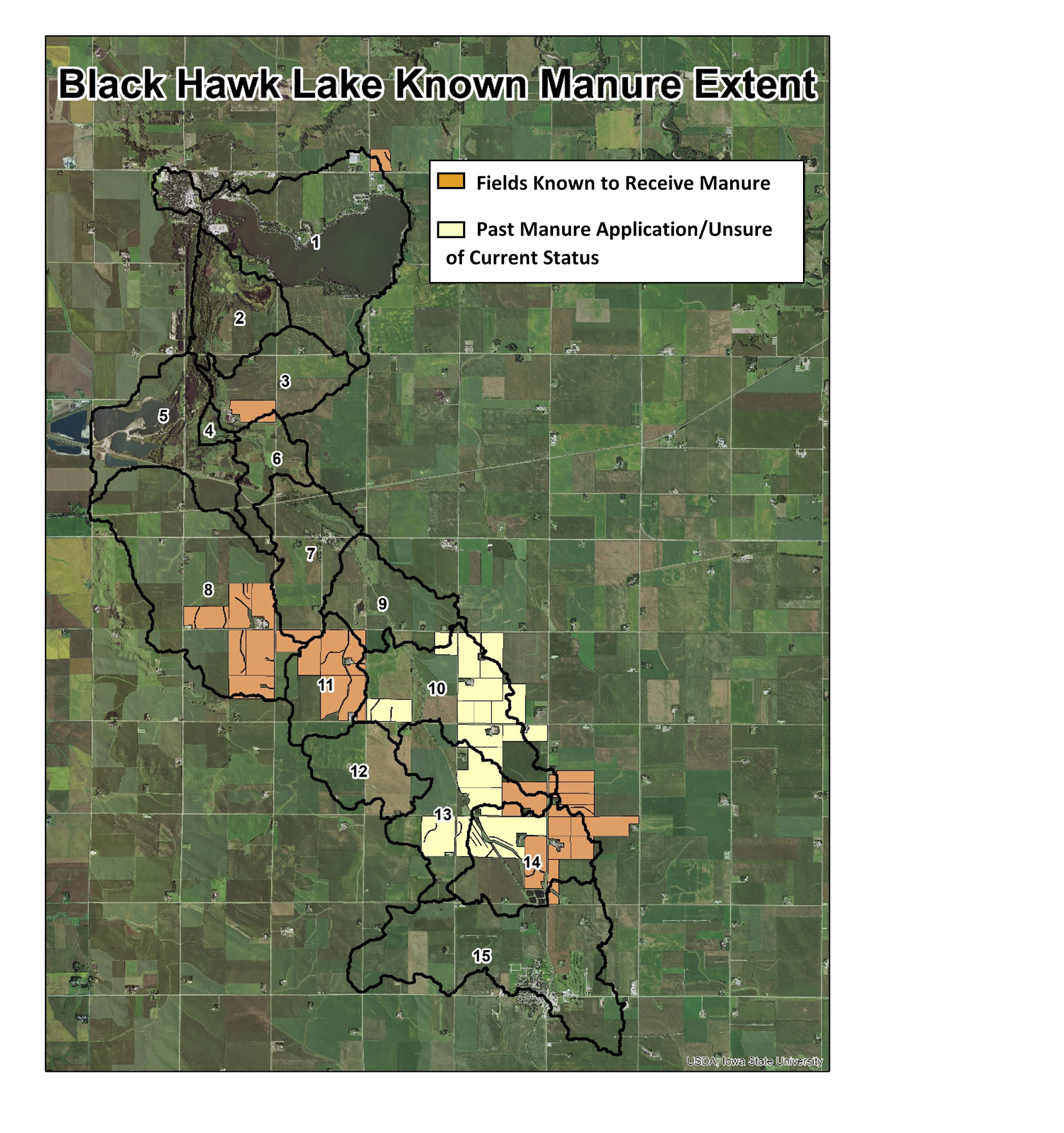

Supplement: S1 Fig — Provided by T.J. Lynn, BHL Watershed Coordinator. (TIF) [file pone.0227136.s001.tif]
