# Supplementary material for: Catchment-scale export of antibiotic resistance genes and bacteria from an agricultural watershed in central Iowa
Source: PLoS One. 2020 Jan 10;15(1):e0227136. doi: 10.1371/journal.pone.0227136 (PMC6953785; doi:10.1371/journal.pone.0227136)
Supplement: S1 Table — *Cropland Data Layer 2018, **Web Soil Survey. (PDF) [file pone.0227136.s002.pdf]

**Property**	**S11**	**ST12**	**T8**	**S15**	**EOC**
Subwatershed area (ha)	229.4	221.2	822.5	727	599.6
Manure Applied (Y/N)	Y	N	Y	N	N
Average slope*	3.5	3.3	3.1	4.6	2.9
BMP implementation (% area of subwatershed)	30.0	87.5	22.5	21.4	x
Land Cover* (% of catchment)					
Row crop (Corn/soybean)	93.6	73.7	71.6	73.6	90.5
Developed	1.0	1.0	1.0	7.9	1.0
**Major subwatershed soil types (%)****					
Canisteo clay loam	3.7	14.6	1.9	9.2	9.6
Clarion loam	47.1	47.9	45.1	34.2	36.1
Coland clay loam	2.5	1.4	17.4	0	0
Nicollet loam	21.4	18.8	16.7	10.7	25.4
Webster clay loam	17.6	11.7	7.3	12.2	14.2
Soils with hydrologic soil groups C&D	25.1	33.0	30.1	53.7	37.9
