# Supplementary material for: Catchment-scale export of antibiotic resistance genes and bacteria from an agricultural watershed in central Iowa
Source: PLoS One. 2020 Jan 10;15(1):e0227136. doi: 10.1371/journal.pone.0227136 (PMC6953785; doi:10.1371/journal.pone.0227136)
Supplement: S2 Table — (PDF) [file pone.0227136.s003.pdf]

	Antibiotic concentration (pg/pocis) n=5					
	No manure			Manure		
	**ST12**			**S11**		
Antibiotic	SMZ	TET	TYL	SMZ	TET	TYL
Average	657	0.11	1,324	1,306	48.8	1,849
standard deviation	556	0.37	1,012	1,028	153	1,494
Median	521	0.00	977	916	0.14	1,110
	**EOC**			**T8**		
Antibiotic	SMZ	TET	TYL	SMZ	TET	TYL
Average	2,333	379	4,023	1,307	117	1,068
standard deviation	3,501	1,199	5,494	948	365	207
Median	547	0.00	1,319	1,208	0.97	1,009
